# Capacity of very noisy communication channels based on Fisher information

**DOI:** 10.1038/srep27946

**Published:** 2016-06-16

**Authors:** Fabing Duan, François Chapeau-Blondeau, Derek Abbott

**Affiliations:** 1 Institute of Complexity Science, Qingdao University, 266071 Qingdao, China; 2 Laboratoire Angevin de Recherche en Ingénierie des Systèmes (LARIS), Université d’Angers, 49000 Angers, France; 3 Centre for Biomedical Engineering (CBME) and School of Electrical & Electronic Engineering, The University of Adelaide, Adelaide, SA 5005, Australia

## Abstract

We generalize the asymptotic capacity expression for very noisy communication channels to now include coloured noise. For the practical scenario of a non-optimal receiver, we consider the common case of a correlation receiver. Due to the central limit theorem and the cumulative characteristic of a correlation receiver, we model this channel noise as additive Gaussian noise. Then, the channel capacity proves to be directly related to the Fisher information of the noise distribution and the weak signal energy. The conditions for occurrence of a noise-enhanced capacity effect are discussed, and the capacity difference between this noisy communication channel and other nonlinear channels is clarified.

It is well known that, for an additive Gaussian noise channel and an energy constrained input signal, the channel capacity can be explicitly calculated^1^,^2^,^3^,^4^. In practical applications, however, communication systems frequently encounter non-Gaussian noise environments, for instance, underwater acoustic noise and low-frequency atmospheric noise^5^,^6^,^7^. Of all channels with power-constrained noise, the capacity of a Gaussian channel is the smallest^1^,^2^,^3^,^4^. Thus, the capacities of non-Gaussian channels are of great interest^5^,^6^,^7^,^8^,^9^,^10^,^11^,^12^,^13^,^14^,^15^. Moreover, from theoretical and practical viewpoints, a very interesting topic is the investigation of the channel capacity with very weak input signals, e.g. deep space communication channels^2^,^9^ and qubit depolarizing channels^15^. A very noisy channel was introduced by Reiffen^8^, and extended by Gallager^2^ and Majani^9^ to model many physical communication channels operating at very low signal-to-noise ratio (SNR). “Very noisy” channels with very low capacity are of significant interest to communications, since Shannon’s theorem guarantees reliable communication as long as the capacity is nonzero^1^,^2^,^3^,^4^,^9^,^16^. Following the approaches developed in^2^,^8^,^9^ and using a power series of characteristic functions, Nirenberg^5^ derived a simple formula of the capacity for the coherent threshold channel with an optimum receiver. For memoryless channels with very weak inputs, Kullback^10^, Verdú^11^ and Prelov^12^ explicitly expressed the asymptotic expressions of the channel capacity closely related to the Fisher information matrix. Recently, Kostal and Lansky^14^ presented an approximate expression for the information capacity in a broad class of discrete-time channels under the constraint of vanishing input amplitude or power, which allows us to analyse the capacity of channels with memory in a convenient way^13^,^14^.

In this paper, under the assumption of low SNR, we will further derive the capacity of a very noisy communication channel, wherein the optimum receiver may be unavailable and noise is not restricted to be white. Based on the central limit theorem, we argue that, for sufficiently large observation times and with the constraint of weak signal energy, the receiver output tends to be Gaussian distributed, and the channel capacity is then computed by a simple formula being directly related to the Fisher information of the noise distribution. We demonstrate the enhancement of capacity via stochastic resonance will not occur in very noisy communication channel with an optimum receiver, but it can occur with generalized correlation receivers suited for practical implementation. Finally, we compare the asymptotic capacity expressions of this noisy communication channel with other capacity formulas in refs 10, 11, 12, 13, 14.

## Results

### Channel capacity for coloured noise

**Figure 1:**
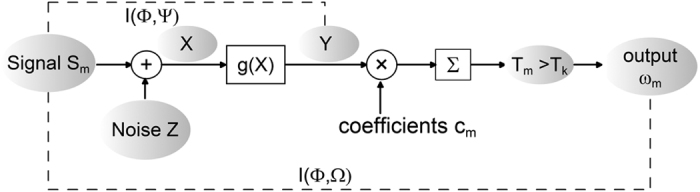
Mutual information *I*(**Φ**, **Ω**) of the communication channel and *I*(**Φ**, **Ψ**) of the nonlinear channel.

For the *M*-ary communication channel shown in Fig. 1, the observation data vector **X** contains the additive noise vector **Z** and the signal vector **S**_*m*_, 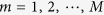. With the assumptions of white noise and very low SNR, Nirenberg^5^ derived the capacity for the coherent threshold channel with an optimum receiver. We briefly present the conclusions of ref. 5 for reference (see **Methods**). However, the idealized assumption of white noise is unpractical, and the coloured noise has practical significance^2^,^3^,^4^. We here further derive a general asymptotic expression of the channel capacity for coloured noise, which applies to not only the optimum receiver but also an arbitrary correlation receiver.

In the case of coloured noise and for very low SNR, the conditional probability function can be expanded to the first order


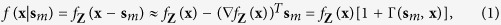


where the operator 
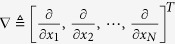
 and the statistic 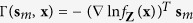. Here, from an information theory point of view by Reiffen^8^ and Gallager^2^, the module 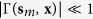 indicates the channel is very noisy in the sense that the channel output is almost independent of the input. For *M* equiprobable signals **S**_*m*_, the receiver takes the maximum likelihood rule


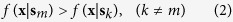


to optimally choose *m*th signal^5^,^17^. Substituting equation (1) into equation (2), the optimum receiver


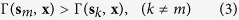


enables us to decide if the *m*th signal was transmitted. For clarity, we state that the statistic Γ(**s**_*m*_, **x**) and the maximum likelihood decoding rule of equation (2) compose an optimum correlation receiver. The channel output is the decoding signal *ω*_*m*_ of the receiver, as shown in Fig. 1.

Then, supposing the zero-mean E_**S**_(**s**) = **0** and extending the very noisy vector channel 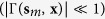
2,5,8,9, the mutual information between the input signal space **Φ** and the channel output space **Ω** is given by


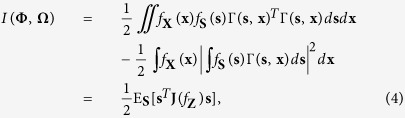


where the Fisher information matrix of the noise distribution is defined as^3^,^7^


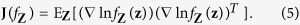


It is noted that **J**(*f*_**Z**_) is also called the Fisher information of a location parameter or the shift-invariant Fisher information^3^,^6^,^7^,^18^, which can be viewed as a special case of the Fisher information measuring the statistical information contained in data about an unknown parameter. Therefore, with the energy constraint of E_**S**_(**s**^*T*^**s**) ≤ *ε* and for the standardized vector 
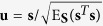
, the channel capacity can be expressed as


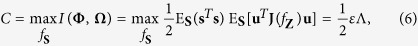


where Λ is the largest eigenvalue of the matrix **J**(*f*_**Z**_) and **u** takes the corresponding eigenvector.

For positive definite matrixes **A**, 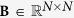 and an arbitrary column vector 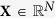, the inequality **X**^*T*^(**A** − **B**)**X** ≥ 0 is abbreviated as 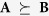. Then, for the positive semidefinite matrix


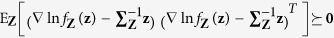


and the noise covariance matrix **∑**_**Z**_ = E_**Z**_(**zz**^*T*^), we have


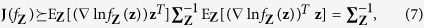


where the equality occurs for *N*-dimensional Gaussian distribution 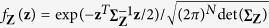 with its Fisher information matrix 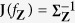. Thus, equation (7) indicates the maximum eigenvalue of 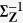 is less than that of Fisher information matrix of non-Gaussian noise. This result extends the conclusion of equation (36) by Nirenberg^5^, and also confirms that, in terms of the channel capacity, zero-mean Gaussian noise is the worst case given that the noise vector has a fixed covariance matrix^3^,^4^.

However, we note the channel capacity of equation (6) is achieved by the optimum receiver of equation (3). In many practical cases, the optimum receiver may be not implementable for the unknown noise distribution or the non-closed form of distributions (e.g. *α*-stable noise^19^). Thus, we further consider the generalized correlation receiver


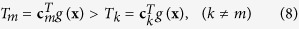


where the coefficient vector 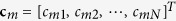 and the function *g*(**x**) is not restricted to be memoryless. For the zero-mean vector of E_**Z**_[*g*(**z**)] = **0** (for a shift in mean)^6^ under *f*_**Z**_ and for very low SNR, *g*(**x**) can be expanded to the first-order


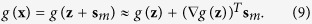


Then, for a large observation size *N*, the statistic *T*_*m*_ has the mean 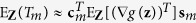 and the variance 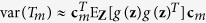. Using the Cholesky decomposition of the symmetrical matrix **V** = E_**Z**_[*g*(**z**)*g*(**z**)^*T*^] = **LL**^*T*^, the output SNR of the receiver can be calculated as


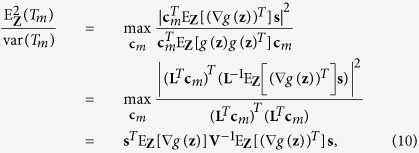


by optimally choosing 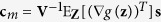. Then, we argue that, for sufficiently large observation times and with the constraint of weak signal energy, the receiver output tends to be Gaussian distributed, and the capacity can be approximately calculated as


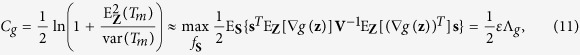


where Λ_*g*_ is the largest eigenvalue of the matrix 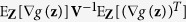. Observing


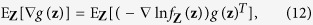


and for the positive semidefinite matrix


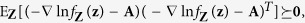


we have


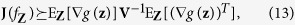


with 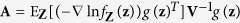 and the equality occurring for 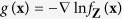. This inequality (13) indicates that the eigenvalue Λ of **J**(*f*_**Z**_) is not less than the eigenvalue Λ_*g*_ of the matrix 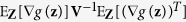. Therefore, based on equations (6), (11) and (13), we find


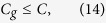


which extends the conclusion of ref. 5 to the case of coloured noise. In addition, the equality in equation (13) also demonstrates the receiver of equation (8) is optimal when 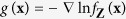, i.e. the optimum receiver of equation (3).

We argue that the asymptotic capacity expression of equation (11) has a broader applicability for an arbitrary correlation receiver operated in coloured or white noise environments. As a simple check for the consistency of the results from equation (11) to equation (14), we consider the case of white noise. Immediately, due to the statistical independence of *g*(**z**), the expectation matrices 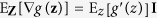 and **V** = E_*z*_[*g*^2^(*z*)]**I**. Here, the derivative *g*′(*z*) = *dg*(*z*)/*dz* and **I** is the unit matrix. Therefore, the matrix 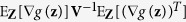 in equation (11) has *N* identical eigenvalues 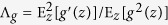, and the channel capacity becomes


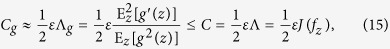


where the eigenvalue Λ = *J*(*f*_*z*_) corresponds to the Fisher information matrix **J**(*f*_**Z**_) in equation (6). Using the Cauchy-Schwarz inequality and integration by parts 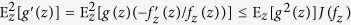, and the equality in equation (15) occurs when 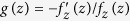 that specifies the optimum receiver in the presence of white noise^5^.

### Conditions for noise-enhanced capacity

Since the emergence of the concept of stochastic resonance^20^, the employment of noise in enhancing the performance of nonlinear systems has become an interesting option^13^,^14^,^21^,^22^,^23^,^24^,^25^,^26^,^27^,^28^,^29^,^30^,^31^,^32^,^33^,^34^,^35^,^36^. Initially, the mechanism of stochastic resonance manifests itself as a time-scale matching condition for the noise-induced characteristic time of systems and the signal period^20^,^27^. Later, the notion of stochastic resonance has been widened to a number of different mechanisms, e.g. aperiodic stochastic resonance^22^ and suprathreshold stochastic resonance^31^. For such stochastic resonance effects^22^,^31^, there is no matching time-scale that corresponds to the input aperiodic or information-carrying random signal, but the system performance still reaches a maximum at an optimal non-zero noise level. Therefore, the noise-enhanced effect, instead of stochastic resonance, becomes a more appropriate term for describing the enhancement effect of system responses via the addition of noise. Here, if the channel capacity reaches a maximum at an optimal non-zero noise level, then the noise-enhanced capacity effect occurs. Otherwise, upon increasing the noise level, the channel capacity monotonically decreases, this is to say, the noise-enhanced capacity effect does not exist.

There are two approaches for varying the noise in stochastic resonance. One is tuning the noise level but not changing the noise type, and the other is adding extra noise to a given noisy signal, while the extra noise type may be different form the original one. Next, we will demonstrate the occurrence or nonoccurrence condition of the noise-enhanced capacity effect by the above mentioned methods.

First, we will prove that no noise-enhanced capacity effect exists for tuning the scaled noise level in an optimum receiver. For the scaled noise vector **Z** = **DZ**_*n*_, the covariance matrix **∑**_**Z**_ can be factored as **∑**_**Z**_ = **DD**^*T*^ and the standardized noise vector **Z**_*n*_ has a covariance matrix being the unit matrix 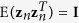
7. A well-known scaling property of the Fisher information matrix is^7^,^18^,^37^,^38^,^39^,^40^


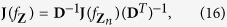


which implies the largest eigenvalue Λ of **J**(*f*_**Z**_) is a monotonically decreasing function of Λ_*n*_/det(**∑**_**Z**_) for the determinants det^2^(**D**) = det^2^(**D**^*T*^) = det(**∑**_**Z**_). Here, the largest eigenvalue of 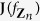 is Λ_*n*_ that is a fixed quantity for **Z**_*n*_. For such a channel with its optimum receiver, equation (11) indicates the channel capacity 
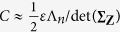
 monotonically decreases as the noise intensity increases. Thus, no noise-enhanced capacity phenomenon will occur by tuning the noise level.

For instance, we consider a threshold receiver based on the function *g*(*x*) = sign(*x*) and the Laplacian white noise with its distribution 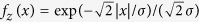. We note that the threshold receiver is optimum for the Laplacian noise, and 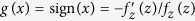 satisfies the equality condition in equation (15). In this case, the channel capacity in equation (15) can be calculated as 
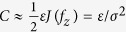
, which monotonically decreases as the noise level *σ* increases. Thus, there is no noise-enhanced capacity effect.

Secondly, we usually have a given signal corrupted by noise, and the initial noise level is unadjustable. We will prove that the addition of extra noise cannot further improve the channel capacity achieved by the optimum receiver. Under this circumstance, we add an extra noise vector **W**, independent of **Z** and **S**_*m*_, to the observation **X**, and the updated data vector is


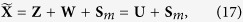


where the composite noise vector **U** = **Z** + **W** with its distribution *f*_**U**_. In this case, we should employ the statistic 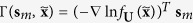 to specify the optimum receiver, and the corresponding capacity is then given by


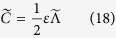


with the largest eigenvalue 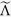 of the Fisher information matrix **J**(*f*_**U**_). For any nonsingular matrix 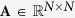, the Fisher information matrix inequality^3^,^37^,^38^,^39^,^40^ holds for


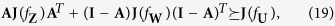


we then find the largest eigenvalue Λ of **J**(*f*_**Z**_) is not less than the largest eigenvalue 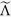 of **J**(*f*_**U**_) and


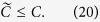


This result of equation (20) clearly shows that stochastic resonance cannot further improve the channel capacity achieved by the optimum receiver, regardless of adding white or coloured noise vector **W**.

**Figure 2:**
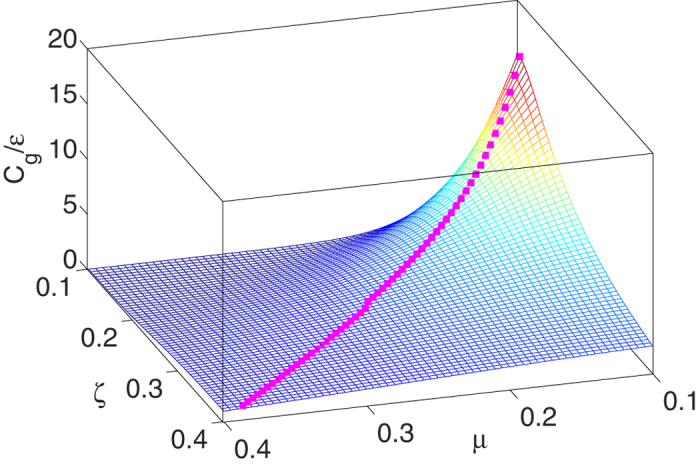
Stochastic resonance effect of the capacity per signal energy *C*_*g*_/*ε* = Λ_*g*_/2 in equation (11) versus the noise parameters *μ* and *ζ* in equation (21). Here, the correlation coefficient *ρ*_1_ = 0.2 and *ρ*_2_ = 0 in the coloured noise model of equation (22). The corresponding maxima of *C*_*g*_/*ε* versus optimal values of *ζ* are also marked by squares.

Thirdly, we note that the above two negative conditions of the noise-enhanced capacity effect arise with the optimum receiver matched to the distribution of the background noise. By Contrast, if the generalized correlation receivers of equation (8) are not optimal for the background noise, stochastic resonance may play an important role in the enhancement of capacity. For example, we consider non-scaled Gaussian mixture noise vector **W** with its distribution^6^,^21^,^28^,^33^


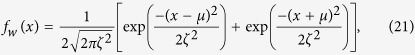


where the variance 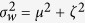 and parameters *μ*, *ζ* ≥ 0. A useful coloured noise model of the first-order moving-average^41^ as


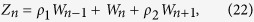


where the correlation coefficients are *ρ*_1,2_ and 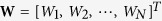 is an independent identically distributed (i.i.d.) random vector. For small values of *ρ*_1,2_ 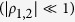, the dependence among noise samples *Z*_*n*_ will be weak^41^. The signum function *g*(*x*) = sign(*x*) is adopted to construct the generalized correlation receiver of equation (8), which is not optimal for the coloured noise **Z**. The optimum receiver indicated in equation (3) for the coloured noise **Z** is rather complicated, since the distribution *f*_**Z**_ does not have a tractable analytic expression^41^. Using the approach developed in ref. 41, we have the expectation matrix


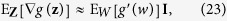


with the unit matrix **I** and 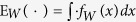, and the matrix **V** becomes tridiagonal with elements


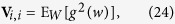



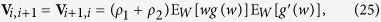


for 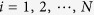, and other elements are higher-order infinitesimal of *ρ*_1_ + *ρ*_2_ 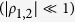. Then, we calculate the largest eigenvalue of the matrix 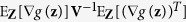 as


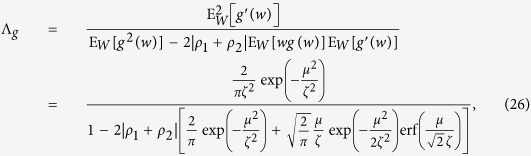


where the error function 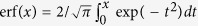. In Fig. 2, we show the capacity per signal energy *C*_*g*_/*ε* = Λ_*g*_/2 in equation (11) versus the noise parameters *μ* and *ζ* in equation (21). Here, the correlation coefficient *ρ*_1_ = 0.2 and *ρ*_2_ = 0 in the coloured noise model of equation (22). We regard the parameters ±*μ* as the peak locations of the Gaussian mixture distribution in equation (21), while the parameter *ζ* as the noise level. It is then clearly shown in that Fig. 2, upon increasing *ζ* for a fixed value of *μ* (the noise variance 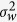 also increases), the noise-enhanced capacity effects exist. The corresponding maxima of *C*_*g*_/*ε* versus optimal values of *ζ* are also marked by squares in Fig. 2.

We emphasize that the above noise-enhanced capacity effect is an illustrative case of stochastic resonance that exists for a suboptimal receiver not matching the background noise. However, this mismatch condition is not the decision criteria for the occurrence of the noise-enhanced effect, since the example illustration is under the assumptions of a small signal and a correlation receiver with a large observation size. Beyond these restrictive assumptions, the noise-enhanced effect has been frequently observed^21^,^24^,^25^,^28^,^29^,^30^,^31^. For instance, the noise-enhanced effect has been demonstrated for non-weak signals in threshold neurons^25^,^29^,^31^, where an optimal matching condition is inapplicable to the neuronal model immersed in complex noisy environments. It is sufficiently recognized that a well-established criterion for the noise-enhanced effect is to observe an optimal noise level at which the system response can be optimized.

## Discussion

In this paper, we analyse the capacity of a very noisy communication channel with correlation receivers. With the weak signal energy constraint and for very low SNR, we generalize an asymptotic expression of capacity achieved by the optimum receivers in a coloured noisy environment. Moreover, for the case when the optimum receiver is unavailable in practice, a capacity formula is presented for the communication channel with a generalized correlation receiver. We further discuss the occurrence condition of the noise-enhanced capacity effect in the considered communication channel.

A similar asymptotic expression of capacity is also obtained in memoryless^10^,^11^ or memory additive-noise channels^12^,^13^,^14^. We emphasize the asymptotic capacity expressions of equations (6) and (11) are different from that in previous literature^10^,^11^,^12^,^13^,^14^. In Fig. 1, for the channel output **Y** = *g*(**X**), these studies assume the conditional probability density as *f*_**Y**|**S**_(**y**|**s**). Then, the Fisher information matrix is defined as^10^,^11^,^12^,^13^,^14^


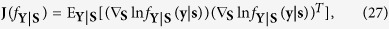


with the operator 
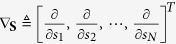
. Then, for the zero-mean signal vector E_**S**_(**s**) = **0** and the weak signal energy *ε*, the mutual information between the input space **Φ** and the output space **Ψ** is approximated as^10^,^11^,^12^,^13^,^14^


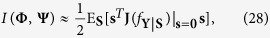


which is different from the mutual information *I*(**Φ**, Ω) of equation (4) based on the Fisher information matrix **J**(*f*_**Z**_) of the noise distribution *f*_**Z**_. It is shown in Fig. 1 that the receiver multiplies nonlinear transformation *g*(**x**) with optimized coefficients, and obtains a cumulative statistic *T*_*m*_ that decides whether the *m*th signal **S**_*m*_ is sent or not. Then, the considered communication channel chooses an optimal signal **S**_*m*_ from the signal space to maximize the average mutual information. Since the receiver collecting the weighted nonlinear outputs as the statistic 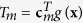, and for any nonlinear function *g*, the distribution of *T*_*m*_ tends to be Gaussian. This leads to the asymptotic expressions of capacity of equations (6) and (11). We recognize the asymptotic capacity expressions in equations (6) and (11) have application in the context of a very noisy communication channel with a correlation receiver. As a new analytical result of the channel capacity, it has theoretical significance and deserves some exposition.

We also note that, for the linear transfer function of **Y** = **Z** + **S**, the conditional probability density *f*_**Y**|**S**_(**y**|**s**) = *f*_**Z**_(**y** − **s**), the Fisher information matrix of equation (27) becomes


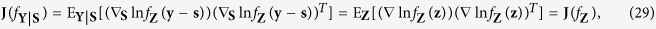


where the differentiation operator 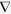 with respect to **S** is equivalent to differentiation with respect to **Z**^3^. Therefore, for the linear additive-noise channel, the considered communication channel has the same capacity as that denoted in refs 10, 11, 12, 13, 14.

Besides a linear channel capacity defined and calculated by Shannon^1^, only a few analytical results exist for a variety of different nonlinear channel models. We argue that our asymptotic capacity expression for a nonlinear channel may be valuable for practical channels and coding techniques developed for communication applications in order to approach the established linear Shannon limit, and deserves further extensive study. We here only consider a single correlation receiver for detecting the weak signal, however recent studies in general provide evidence that, besides an optimal noise intensity, an optimal network configuration exists, at which the best system response can be obtained^22^,^31^,^42^,^43^,^44^,^45^,^46^. Thus, an interesting extension for future work is to investigate the capacity of a very noisy communication channel with receivers connected in various network configurations.

## Methods

### Very noisy communication channel model

Consider a coherent *M*-ary communication channel transmitting *M* possible signals **S**_*m*_ for 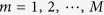, as shown in Fig. 1. In an interval, the observation vector


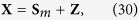


where 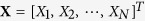 contains the noise vector 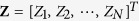 and the signal vector 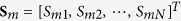. Then, a receiver multiplies the transformation *g*(**X**) with optimized coefficients, resulting in a cumulative statistic *T*_*m*_(**X**) for deciding whether the *m*th signal **S**_*m*_ is sent or not. The capacity *C* of a communication channel is given by the maximum of the mutual information *I*(**Φ**, **Ω**) between the input signal space **Φ** and the channel output space **Ω**


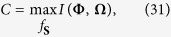


where the maximization is with respect to the input distribution *f*_**S**_ over the signal space **Φ**^1^,^2^,^3^,^4^,^5^.

### Nirenberg’s approach for white noise

The white noise **Z** has the multivariate distribution 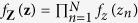 with zero-mean and variance 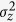. Let the statistically independent signal components be constrained to satisfy 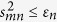, and the total signal energy has a constraint 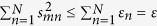. Then, for very low SNR of 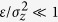, the conditional probability density can be approximated as


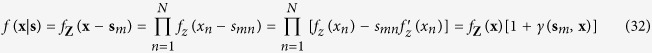


with the first two terms of Taylor series. Here, 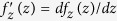 and the statistic 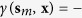

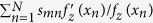
5. Using the maximum likelihood rule^17^, the conditional probability density on the knowledge that the *m*th signal satisfies


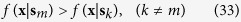


which leads to the optimum receiver


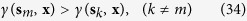


to decide if the *m*th signal was sent.

To simplify the mathematical manipulations, Nirenberg^5^ assumes the even noise distribution function *f*_*z*_(*z*) = *f*_*z*_(−*z*) and a very noisy channel 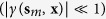
2,8,9 yielding the mutual information between the output space **Ω** and the input signal space **Φ** as


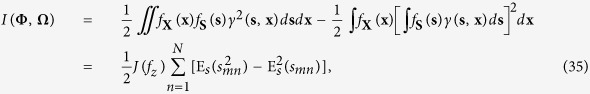


where the Fisher information 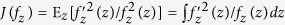 of the noise density *f*_*z*_ and the expectation 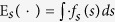. Since the same bias 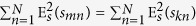 does not affect the decision of inequality of equation (34), it may be conveniently assumed to be zero^5^. Then, over the class of signal distributions *f*_**S**_, the channel capacity is computed as^5^


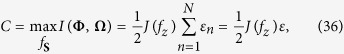


which is applicable to various white noise types.

Furthermore, for a fixed noise variance 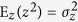 and an arbitrary noise density function *f*_*z*_, 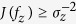
17, where the equality occurs for Gaussian distribution 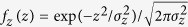 with its Fisher information 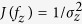
7,18,37. Accordingly, the additive Gaussian noise channel is the worst one, and has the minimum capacity, as indicated in equation (36). It is well known that, for very low SNR of 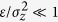, the capacity of Gaussian vector channel is approximately calculated as^2^,^3^,^4^


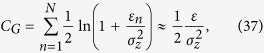


which accords well with equation (36)^5^.

## Additional Information

**How to cite this article**: Duan, F. *et al.* Capacity of very noisy communication channels based on Fisher information. *Sci. Rep.* **6**, 27946; doi: 10.1038/srep27946 (2016).
